# The Saliva, from a Dental Standpoint

**Published:** 1893-02

**Authors:** Carrie M. Stewart


### The Saliva, from a Dental Standpoint.

BY CARRIE M. STEWART, D.D.S.

This fluid is one with which everyone is familiar, performing, as it does, the lubrication of the oral cavity and assisting in the processes of deglutition and digestion.

As it appears in the mouth, the saliva is inodorous, transparent, and, in the healthy individual, of an alkaline reaction.

There is a variation in the consistency of the fluid, according to the gland secreting it; that from the parotid gland being clear and limpid, the first few drops secreted often being acid in character.

Upon standing, a deposition of calcium carbonate takes place.

The submaxillary saliva is viscid in character and contains the salivary corpuscles found in the mixed saliva.

That secreted by the sublingual gland is more viscid and richer in salts than the sub-maxillary secretion.

As found in the mouth, the fluid consists of a mixture of the saliva from the various glands, and is sufficiently alkaline to neutralize any acidity of the oral mucous.

As it issues from the glands, saliva is germ free, but in the mouth it takes up various foreign substances, including bacteria, from the deposits which may be present upon the teeth.

The action which this fluid has upon the starches of the food is quite well understood, but its effects upen the teeth and adjacent structures have not been satisfactorily demonstrated.

It has been given the credit of producing chemical abrasion of the teeth, when in a vitiated condition, but this question has not been definitely settled as yet.

The calcareous deposition from saliva enters largely into the composition of salivary calculus, and the green discoloration of enamel is found, most frequently, in patients having abnormally viscid saliva.

Acidity of saliva may cause increased redness and irritability of the mucous membrane of the mouth, with excoriations and bleeding of the gums. On the other hand these diseased conditions may produce an acidity in saliva which would otherwise be normal in reaction.

Saliva acts as an irritant under certain conditions, and increases the diseased condition by its presence.

It might be well in many cases, which seem to baffle all attempts to cope with them, to test the reaction of the saliva with a neutral litmus solution, to ascertain whether it may not be that the vitiated saliva is playing an important part in the cause of the disease.

Where no actual disease of the mouth is present, the saliva may, from some cause, be acid. Especially is this true in cases of chronic dyspepsia, and the presence of a constantly acid fluid in the mouth is anything but beneficial to the teeth.

The diseases occurring in the mouth will be better understood and more intelligently treated, when due consideration is given to the action of saliva upon the oral tissues, especially when the fluid is in a vitiated condition.

